# Apoptogenic Metabolites in Fractions of the Benthic Diatom *Cocconeis scutellum parva*

**DOI:** 10.3390/md12010547

**Published:** 2014-01-22

**Authors:** Valerio Zupo, Friedrich Jüttner, Chingoileima Maibam, Emanuela Butera, Judith F. Blom

**Affiliations:** 1 Functional and Evolutionary Ecology Laboratory, Stazione Zoologica Anton Dohrn, Punta San Pietro, Ischia 80077, Italy; E-Mails: chingoileima.maibam@szn.it (C.M.); emanuela.butera@szn.it (E.B.); 2 Limnological Station, University of Zurich, Seestrasse 187, Kilchberg CH-8802, Switzerland; E-Mails: juttner@limnol.uzh.ch (F.J.); blom@limnol.uzh.ch (J.F.B.)

**Keywords:** *Cocconeis*, *Hippolyte*, fractionation, apoptosis, cancer, diatom

## Abstract

Benthic diatoms of the genus *Cocconeis* contain a specific apoptogenic activity. It triggers a fast destruction of the androgenic gland in the early post-larval life of the marine shrimp *Hippolyte inermis*, leading to the generation of small females. Previous *in vitro* investigations demonstrated that crude extracts of these diatoms specifically activate a dose-dependent apoptotic process in human cancer cells (BT20 breast carcinoma) but not in human normal lymphocytes. Here, a bioassay-guided fractionation has been performed to detect the apoptogenic compound(s). Various HPLC separation systems were needed to isolate the active fractions, since the apoptogenic metabolite is highly active, present in low amounts and is masked by abundant but non-active cellular compounds. The activity is due to at least two compounds characterized by different polarities, a hydrophilic and a lipophilic fraction. We purified the lipophilic fraction, which led to the characterization of an active sub-fraction containing a highly lipophilic compound, whose molecular structure has not yet been identified, but is under investigation. The results point to the possible medical uses of the active compound. Once the molecular structure has been identified, the study and modulation of apoptotic processes in various types of cells will be possible.

## 1. Introduction

A variety of natural compounds with medical applications have been found in marine macro-algae [1,2], whereas the study of secondary metabolites from micro-algae and their biotechnological applications remains in its infancy [3,4]. Chemical ecology investigations may help find compounds with defined bioactivity [5,6] present in various diatoms, in particular those derived from the oxylipin pathway [7,8,9]. However, these studies are mostly related to planktonic organisms, often aimed at determining possible effects of diatoms on human health [10,11]. Very limited information is available about bioactive compounds produced by benthic diatoms and their biotechnological applications [12,13].

**Figure 1:**
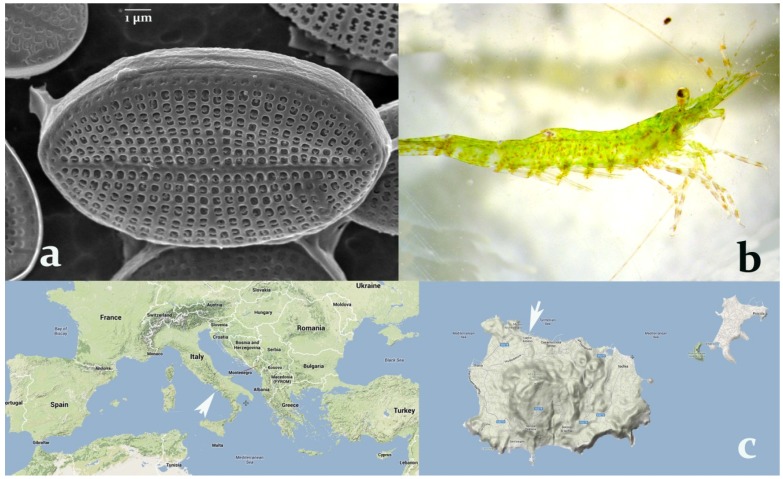
(**a**) *Cocconeis scutellum parva* observed under the SEM (7000×). (**b**) *Hippolyte inermis*, young male. (**c**) Sampling area for both the shrimp and the diatom (40°45′00″N 13°53′00″E), indicated by white arrows, at the Island of Ischia (right), in the bay of Naples, Italy (left).

The diatoms of the genus *Cocconeis* exhibit a peculiar biological activity effective on marine shrimps. In particular, *C. scutellum parva* (Figure 1a), seasonally present on the leaves of *Posidonia oceanica* and particularly abundant in spring, was demonstrated to induce an early shift to the female sex [14] in the shrimp *Hippolyte inermis* Leach (Figure 1b), living in the same environment. Still unknown compound(s) trigger a rapid apoptosis of the androgenic gland (AG) of this shrimp, followed by sex reversal from male to female [15]. The strong influence of food on the sex of an invertebrate has been demonstrated only in this predator–prey relationship, and it is fundamental to assure stability to natural populations of this shrimp [16].

The proterandric sex reversal is a natural process in this species, occurring approximately after the first year of the shrimp life [17]. However, it has been shown [18] that the presence of ovigerous females would be continuously decreasing if based only on sex-reverted specimens (older than one year). In spring, the feeding on specific diatoms induces the production of early-developed females (small individuals, named *Beta* females) that contribute to the autumn reproductive season. Therefore, the apoptotic destruction of the AGs in males of *H. inermis*, triggered by the feeding on particular species of diatoms, represents a stabilizing factor for natural populations [14], since it increases the abundance of ovigerous females during the autumn reproductive season. This process is possible because the sex of crustaceans is determined by the presence/absence of a single gland, the AG [19], producing an insulin-like hormone that prompts the development of male characters [20]. The silencing of this hormone, prior to the appearance of male sexual characteristics, caused the full and functional sex reversal of males into neo-females, in the giant river prawn *Macrobrachium rosenbergii* [21]. Similarly, the ablation of the AGs induces a shift to the female sex in various crustaceans [22,23].

Specifically, in the case of *H. inermis*, the peculiarity of this process is in the selectivity of the action: A few species of *Cocconeis* diatoms [24] are active only on the small AG gland of this shrimp, in a very narrow time window (from the second to the twelfth day of post-larval life). The same species of diatoms were tested in other times [15] or on other decapods [25] but they did not elicit any bioactivity comparable to the one detected on young post-larvae of *H. inermis*. Additionally, toxicity tests performed with crude extracts of the diatom *C. scutellum parva* on embryos of the sea urchin *Paracentrotus lividus* did not elicit any significant effects [26]. Consequently, it may be worth identifying the metabolite responsible for the peculiar activity of these diatoms [27], characterized by a highly selective apoptogenic power, in order to develop new natural drugs useful for human anticancer therapies [6].

Programmed cell death (apoptosis) influences several biological processes in multi-cellular organisms [28], and it is involved in various diseases, including cancer [29]. The lack of activation of apoptosis leads to the proliferation of neoplastic cells in several organisms and most anti-cancer drugs currently used trigger the apoptosis of various types of cells [30], although their main disadvantage is represented by the wide spectrum of tissues targeted in the recipient body [31].

Previous research [32] demonstrated that apoptosis is triggered in human breast cancer cells (BT20 cells), *in vitro*, by the administration of very low doses of lipophilic fractions of the diatom *C. scutellum parva*, through the activation of caspase-8 and caspase-3, the initiator proteases involved in the extrinsic pathway, blocking the progression of the cell cycle from S to G2-M phases [33]. In contrast, the intrinsic pathway, mediated by caspase-9 [34], is not activated by *C. scutellum* extracts in cultured cancer cells. This demonstrates that the activation promoted by the diatom is not due to a generic toxicity [35], but it is due to the specific activation of cell surface apoptosis-inducing ligands [36].

Several marine algae have been demonstrated to produce apoptogenic compounds that could be promising candidate drugs for cancer therapy [37]. However, all of them exhibit a generic wide spectrum of activity, inducing damage also in normal cells [38]. For this reason, the research of the specific apoptogenic compound contained in crude extracts of *C. scutellum parva* is promising.

Since the shrimp is so far the only biological “sensor” able to track the presence of the active compound(s), each new fraction obtained has to be tested on the shrimp’s larvae in order to detect the narrowest fraction containing the active compound, able to trigger the selective destruction of its AG. The research is complex because the compound is active at very low concentrations (0.1–10 ng of the extracted diatom biomass/mL), is likely present in very small amounts in the wounded cells, and is masked, as found during the HPLC analyses, by several other lipophilic compounds present in larger abundance in diatom cells.

In addition, the shrimp exhibits a single reproductive period every year, with two main peaks of recruitment [39]. Therefore, the production of new fractions and bioassays on shrimp post-larvae must be conducted yearly, since the research is limited by the field availability of ovigerous females. We report here the results of six years of bioassay-guided investigations, leading to the purification and characterisation of the active fraction produced by diatoms collected in Ischia (Italy; Figure 1c).

## 2. Results

### 2.1. Fractionation and Bioassays

**Figure 2:**
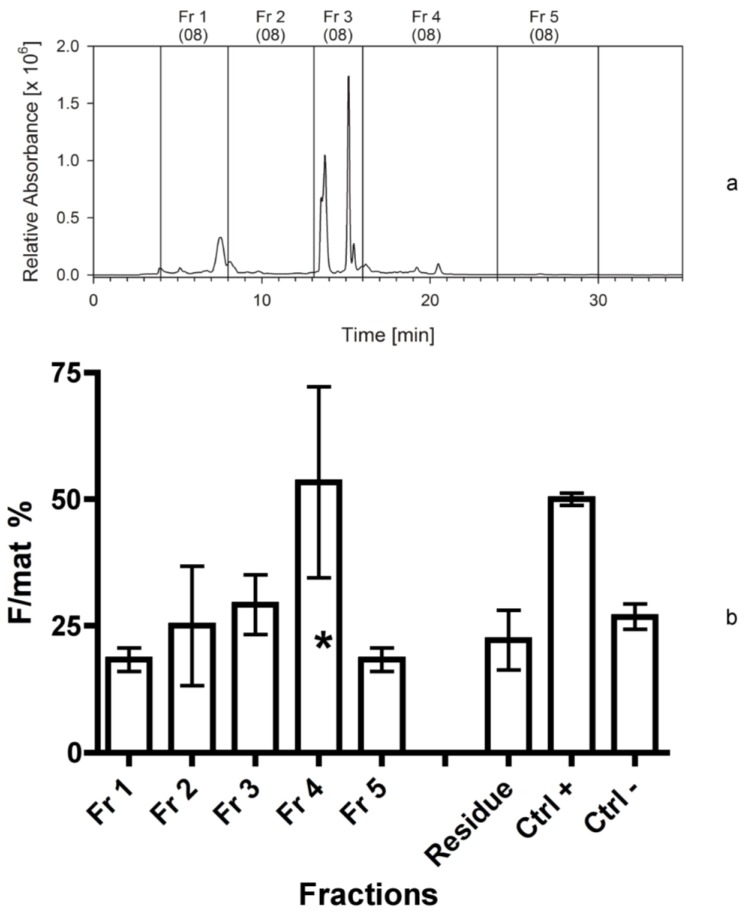
(**a**) Separation of *Cocconeis scutellum parva* extract, performed in September 2008. HPLC chromatogram at 665 nm with the marked fractions; numbers in brackets indicate the year the separation was made. (**b**) Efficacy of the five fractions and of the residue, as indicated, as percentage of females on the total of mature individuals (F/mat%). An asterisk indicates significant differences between treatments and the negative controls.

Previous metabolite profiling [40] and VOC analysis [41] of *C. scutellum parva* demonstrated a large number of low-weight molecular compounds, though bioassays indispensable to trace the active fraction(s) were not conducted. In this study, the residue, composed of proteins, polymeric carbohydrates and silicate frustules, exhibited no activity when added to the food of *H. inermis* (Figure 1b and Figure 2b). This evidence demonstrated that macromolecular compounds were not responsible for the induction of sex reversal. Therefore, the soluble part was separated by HPLC and divided into several fractions. These fractions covered the whole spectrum, from hydrophilic to extremely lipophilic components. The bioassays showed that two major activities were present, one among the hydrophilic compounds and another among the lipophilic compounds. Since in most experiments the lipophilic part was more active than the hydrophilic, we first characterized the lipophilic bioactive fraction and studied it in detail.

The first separation resulted in five fractions (Figure 2a). Fraction 3 was green and contained the highest amount of chlorophyll *c* and derivatives of chlorophyll *a* as indicated by the absorption spectra. The highest activity in terms of sex reversal was measured in fraction 4, with an F/mat% value of 53% ± 19%. *Z*-test indicated significant differences of this fraction, compared to the negative control (*p* < 0.01). The solid cell residue, in contrast, showed absence of activity. The bioassay indicated a skewed distribution of the activity in the fractions, with a minimum in fraction 1 and a maximum in fraction 4 (Figure 2b). For this reason, the next bioassay focused on further separations of fraction 4.

**Figure 3:**
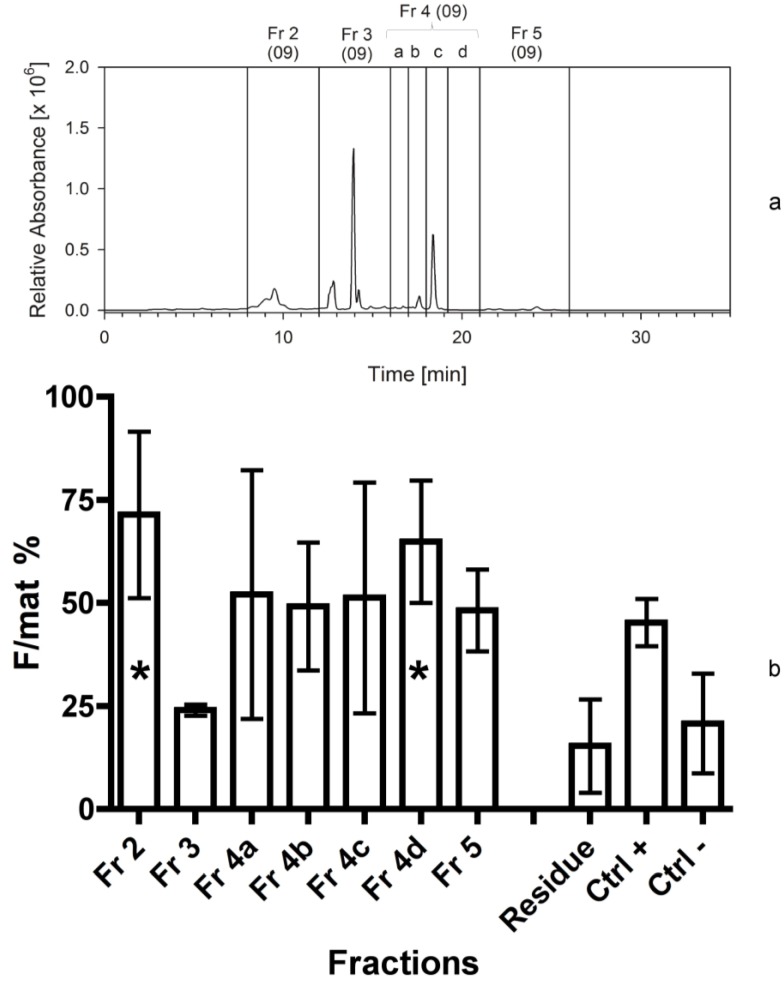
(**a**) Separation of *Cocconeis scutellum parva* extract performed in April 2009. HPLC chromatogram at 665 nm with the marked fractions; numbers in brackets indicate the year the separation was made. (**b**) Efficacy of the 7 fractions and of the residue indicated as percentage of females on the total of mature individuals (F/mat%). An asterisk indicates significant differences between treatments and the negative controls.

The main result of the second bioassay was the finding that the sub-fraction 4d (Figure 3a) exhibited the highest bioactivity of the lipophilic compounds (F/mat% 65 ± 15). An additional high activity was found in the hydrophilic part represented by fraction 2 (F/mat% 71 ± 20). Fraction 3, which contained the highest amount of chlorophylls, exhibited no activity. The solid residue was confirmed to be totally inactive (Figure 3b).

**Figure 4:**
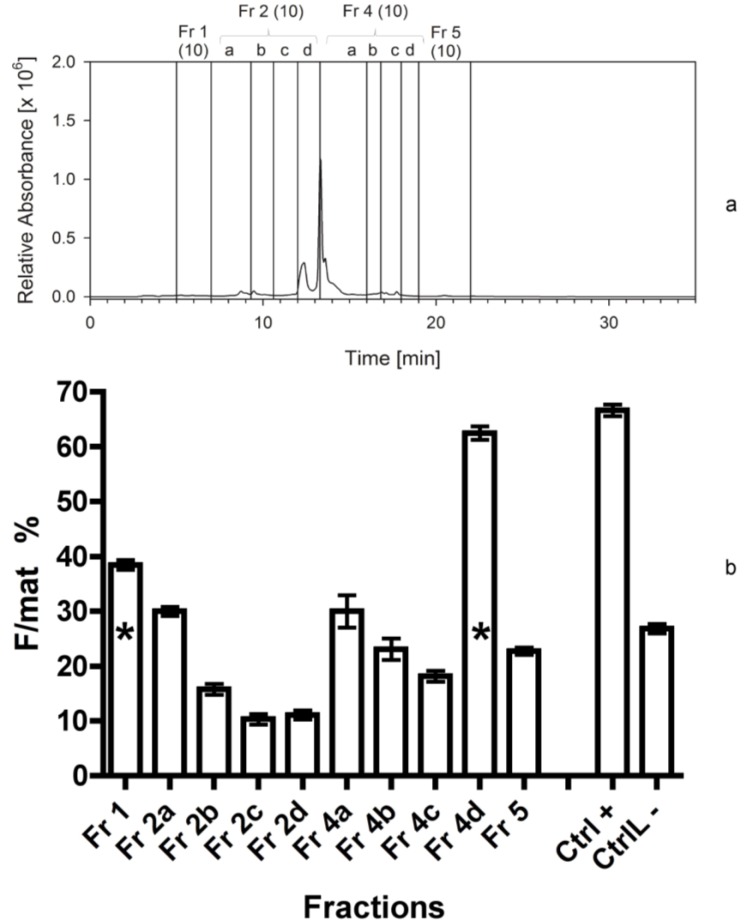
(**a**) Separation of *Cocconeis scutellum parva* extract performed in September 2010. HPLC chromatogram at 665 nm with the marked fractions; numbers in brackets indicate the year the separation was made. (**b**) Efficacy of the 10 fractions and of the residue indicated as percentage of females on the total of mature individuals (F/mat%). An asterisk indicates significant differences between treatments and the negative controls.

In the next separation, fractions 2 and 4 (exhibiting the highest bioactivity in former bioassays) were further separated into smaller sub-fractions (2a–d; 4a–d). The highest bioactivity of the third bioassay was again found in the fraction 4d (F/mat% 63 ± 1), which was nearly as high as the positive control (Figure 4a,b). The fractions 1 and 2a turned out to show medium bioactivity in the hydrophilic part of the chromatogram, with fraction 1 significantly higher than negative controls. Some fractions produced even a lower effect than the negative controls but the difference, in this case, was not significant (*Z*-test, *p* > 0.05).

**Figure 5:**
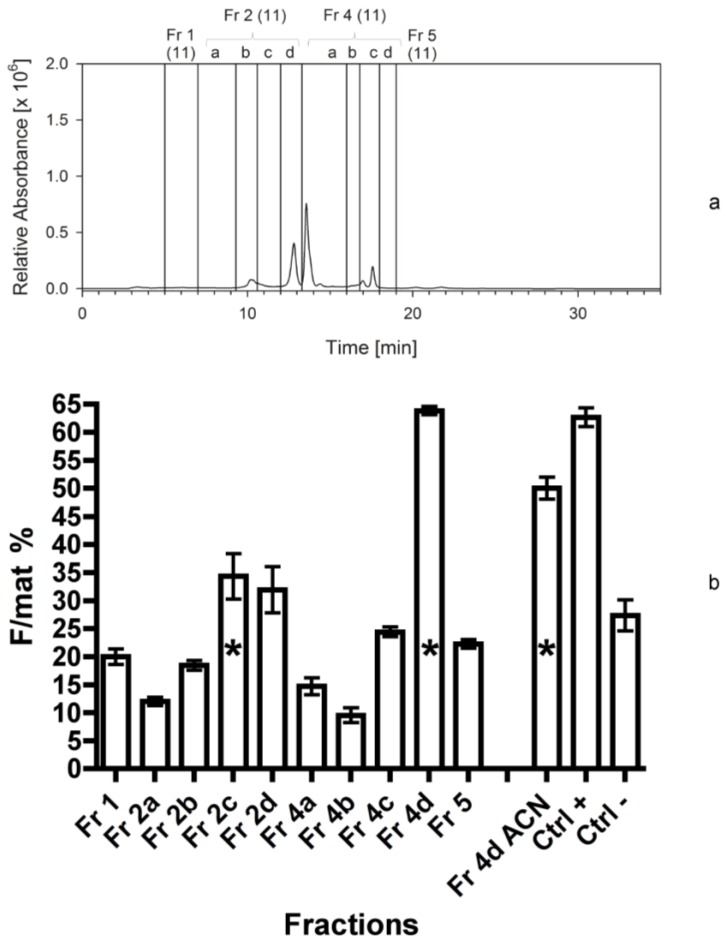
(**a**) Separation of *Cocconeis scutellum parva* extract performed in September 2011. HPLC chromatogram at 665 nm with the marked fractions; numbers in brackets indicate the year the separation was made. (**b**) Efficacy of the 10 fractions and of the ACN solved residue of fraction 4d, indicated as percentage of females on the total of mature individuals (F/mat%). An asterisk indicates significant differences between treatments and the negative controls.

The fourth bioassay was a repetition of the former separation and bioassay (Figure 5a). Again, fraction 4d (Figure 5b) turned out to have a bioactivity as high as the positive control (F/mat% 64 ± 1). A significant effect was produced by fraction 2c, as well. Also in this case some fractions exhibited an activity that was lower than negative controls, according to the natural variability inducing the presence of small amounts of females in any natural population.

Normally, the dried extracts were taken up in 100% methanol, and added to the food particles for the bioassays. However, since the activity could not be completely dissolved in methanol, we solved again the methanol-extracted residue in the vial of fraction 4d with acetonitrile (ACN). Considerable activity was found also in the second solution (fraction 4d ACN; F/mat% 50 ± 1; Figure 5b).

### 2.2. Final Separation of the Bioactive Compound

The knowledge of the physical behaviour of the lipophilic bioactive fraction was used to introduce a new version of pre-concentration and to modify the HPLC separation. Such a pre-concentration is advantageous because higher amounts of the extracts can be administered to one HPLC separation. To allow diode array detection in the UV-C region, the separation was performed with isocratic conditions using solvents (acetonitrile and water) with low absorption in the range between 190 and 250 nm.

**Figure 6:**
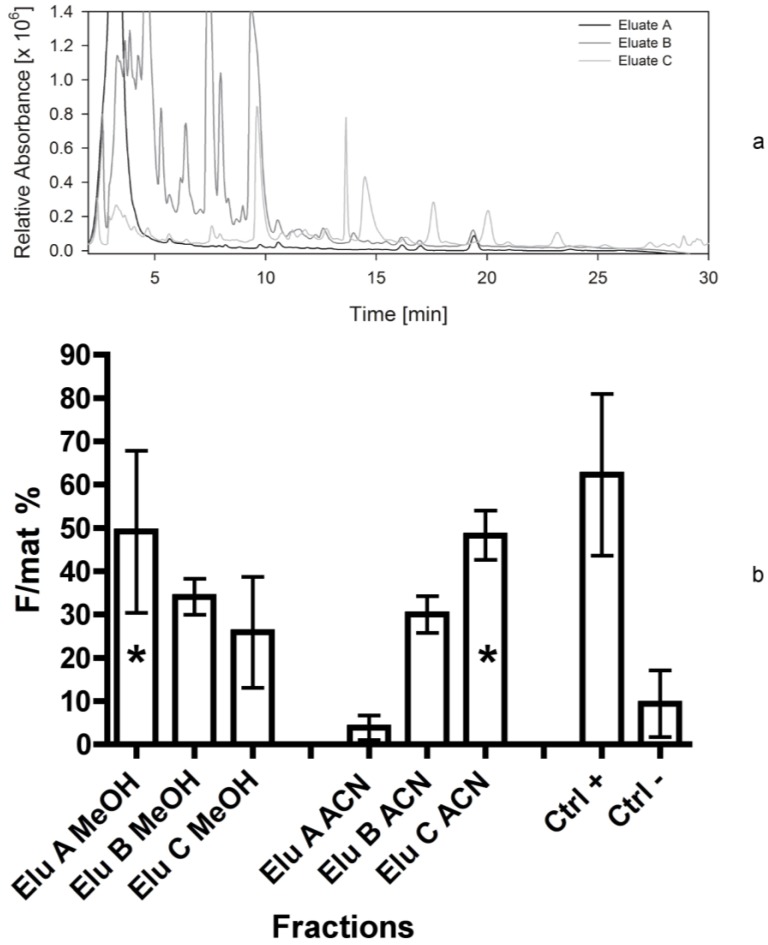
(**a**) HPLC chromatograms (at 195 nm) of eluates A, B, and C of *Cocconeis scutellum parva*. (**b**) Efficacy of the C18 cartridge eluates indicated as percentage of females on the total of mature individuals (F/mat%) of the C18 cartridge eluates, after dissolving in methanol (MeOH) or acetonitrile (ACN), as demonstrated by the fifth bioassay. An asterisk indicates significant differences between treatments and the negative controls.

The diatom extract was subjected to a pre-separation on a C18 cartridge and eluted with 10% ACN (eluate A), 80% ACN (eluate B) and 100% ACN (eluate C), prior to analysis by HPLC (Figure 6a). The eluate C contained all the indicator absorption peaks in the lipophilic region (chromatogram at 195 nm). The bioassay confirmed the chromatographic data and showed the highest bioactivity in eluate C (Figure 6b). In this case the three dried eluates were dissolved twice (as described in the experimental section), in methanol and then in acetonitrile, prior to their addition to experimental foods, and the bioassay indicated that the highest activity in the methanol-dissolved eluates is due to the eluate A (F/mat% 49.14 ± 18.69), while in the ACN-dissolved eluates it is due to the eluate C (F/mat% 48.28 ± 5.66). Both demonstrated significant differences towards the negative controls (Figure 6b), while eluate A dissolved in ACN exhibited an effect lower, but not significantly different in respect to negative controls.

**Figure 7:**
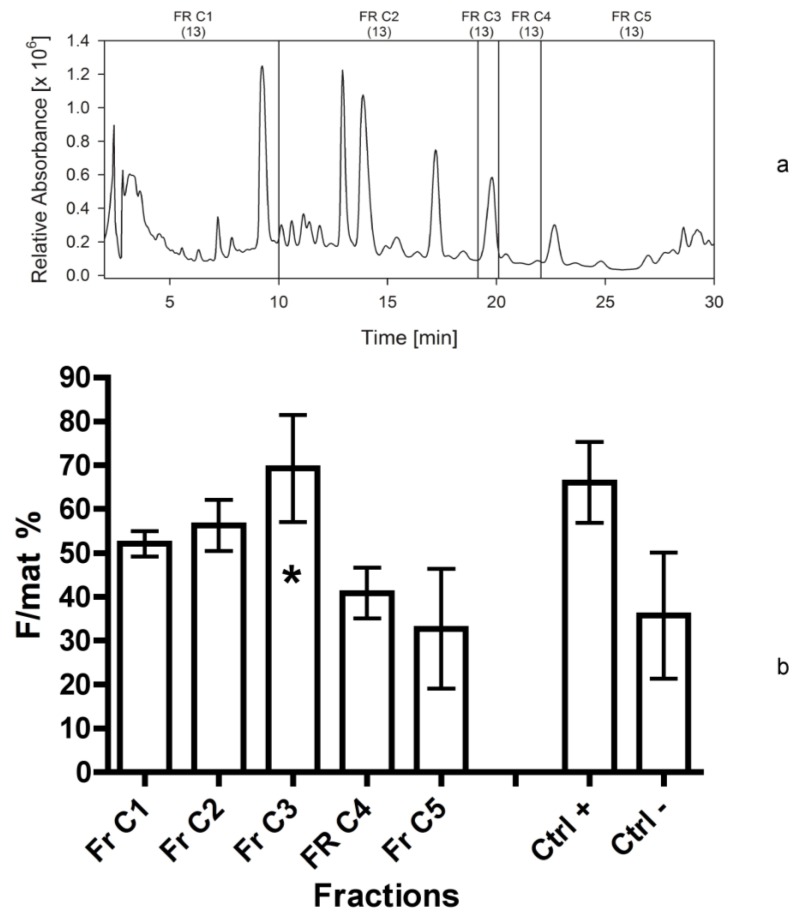
(**a**) Separation of eluate C in April 2013. HPLC chromatogram at 195 nm with the marked fractions of *Cocconeis scutellum parva*; numbers in brackets indicate the year the separation was made. (**b**) Efficacy of the five fractions and of the residue indicated as percentage of females on the total of mature individuals (F/mat%). An asterisk indicates significant differences between treatments and the negative controls.

In the final separation, eluate C was used for further sub-fractionation of the lipophilic area (Figure 7a). After comparing fraction 4d obtained for the third and the fourth bioassays, under the same isocratic conditions as applied for the eluates A, B and C, the peak eluting at 19.8 min (which exhibited a maximum absorption at 197 nm) was chosen as a possible bioactive compound and collected separately (Figure 7a). In fact, both fractions 4d consistently showing high bioactivity exhibited no absorption in the range between 250 and 800 nm.

We therefore tested in the last bioassay five fractions derived by the eluate C. The bioassay demonstrated (Figure 7b) that fraction C3 (dissolved in acetonitrile in the last bioassay), consisting of this single peak, exhibited the highest activity on *H. inermis* (F/mat% 70 ± 12) and its activity was significantly different in respect to negative controls.

## 3. Discussion

The bioactive lipophilic compound eluted from the HPLC column after the chlorophyll derivatives exhibited no absorption in the visible light spectrum. Applying isocratic low-light-absorbing solvents for separation, an absorption maximum of the active compound at 197 nm was observed by diode array detection. This evidence rules out compounds that contain functional groups with features of a chromophore. Such a conclusion is supported by the high lipophilicity of the compound. A high lipophilicity is also supported by the observation that MeOH is a much weaker solvent for the bioactive compound than ACN. Experiments have shown that the compound is very stable. Inactivation, due to storage or treatments, has not been observed. However, the ratios of activity between hydrophilic bioactive fraction and the lipophilic fraction varied in the batches tested. Therefore, we assume that hydrolytic processes may convert a hydrophilic cellular constituent by abstraction of a hydrophilic molecular part into a lipophilic compound. The lipophilic part would be responsible for the apoptotic activation while the hydrophilic part modulates the transport of the molecule. It is possible that such a reaction already takes place during the lyophilisation process or the subsequent extraction, as shown for the generation of free fatty acids [42]. However, also two structurally not related compounds could be present, since it was not yet shown that the isolated bioactive hydrophilic fraction may be converted into a bioactive lipophilic compound.

The bioassays following the first separation immediately indicated that fraction 4 contained a high activity and it could be a good candidate for further separations. The assays also demonstrated that the residue of the extraction, constituted by broken frustules and cell organelles, did not contain any apoptogenic activity. Therefore, the solvents used for the extraction and the techniques applied were effective and, since the second bioassay, it appeared clearer that two fractions were active, the first (fraction 2) more hydrophilic than the second (fraction 4d). This evidence might be due as well to a modification of a single compound during its bio-production process, which does not modify the primary active group. In addition, it was clear from the HPLC chromatograms that the apoptogenic compound could be present in small amounts and be masked by other compounds present in large abundance but with no bioactivity on the shrimp.

In fact, plots were dominated by chlorophylls and other pigments, peaking in the fraction 3, but also present in other fractions. All subsequent fractionations confirmed the positive results for fraction 4d, but the activity appeared to be shared by other fractions characterized by a higher polarity, in the range of fractions 1 and 2a. This activity was probably due to compounds characterized by narrow peaks in the fractions, which may be present in the last part of fraction 1 or in the first part of fraction 2a. Therefore, the compounds present in fraction 4d appear more stable and consistently positioned, while the biological activity of alternative compounds, present in the first eluates, is quite variable (maybe due to a variable level of esterification).

The presence of two compounds exhibiting comparable apoptogenic activity should not be a surprise since there are various apoptogenic compounds produced by marine diatoms [13], bearing a similar basic structure but being variously modified within diatom metabolic pathways [43], as, for example, galacto-pyranosyl-sn-glycerol, epoxy-eicosa-tetraenoic acid, hydroxyl-epoxy-eicosa-tetraenoic acid. In the latter case, for example, hydroxylation of a glycerol chain places compounds with different hydrophilicity but similar functions into groups [44].

Interestingly, when the vessel of the fraction 4d in the last bioassay was refilled with acetonitrile, an additional activity was detected. This demonstrates that the compound is highly lipophilic and that it is not totally soluble in methanol. The high activity exhibited both by fraction 4d in methanol and its further dissolution in acetonitrile also demonstrated that it is active at very low dosage: Even the residue compound still adherent to the vessel after the rinsing with methanol is able to trigger an evident biological activity. The active compound should not be a volatile organic compound (VOC) since these compounds, accurately investigated [41] in the same strain of *C. scutellum parva*, have been largely removed by repeated evaporations, according to the protocols used for the extraction and incorporation in experimental foods.

*Cocconeis scutellum parva* contains a large number of mono-, di- and tri-unsaturated aldehydes with chain lengths from C_5_ to C_10_ [41] and some saturated aldehydes (heptanal, octanal, nonanal, decanal, undecanal), but it contains also fatty acid cleavage products of C_5_ and C_8_ chain lengths (aldehydes, vinyl alcohols and diones). Therefore, given the results of HPLC separations, the active compound might be a modified fatty acid (possibly in the most abundant categories of C_5_ or C_8_) with a peculiar functional group. Other investigations demonstrated the importance of the oxylipin pathways in various diatoms [13] and we know that polyunsaturated aldehydes (PUA) may be toxic and induce apoptosis and necrosis in the embryos deriving from females of marine copepods feeding on these microalgae [45]. In that case, however, the apoptosis was non-specific and it followed the destruction of various tissues as a result of their toxicity [46]. Our compound has a very specific apoptogenic activity and it was demonstrated to be non-toxic [26] when tested on sea urchin embryos. Therefore, our candidate compound might be related to the fatty acid pathway [47], be non-volatile [10] and non-toxic [48], and it could be characterized by a C_5_ or C_8_ chain length [41], probably involved in the mechanism of chemical defence of this diatom.

The chemical defences of marine algae are quite complex and highly evolved. Many of them have been related to invertebrate reproductive failures [13]. The ingestion of diatoms by copepods has been suggested as being detrimental for nauplii hatching success [49]. Also dinoflagellates inhibited the hatching success and larval survival of the scallop *Chlamys farreri* [50], and *Heterosigma carterae* was made responsible for suppressing the egg-hatching rates of the copepod *Acartia tonsa* [51]. In terms of reproductive impacts, the oxylipins are of great interest. They are produced in diatoms from fatty acids, mainly after wounding of the cells, and include (poly)unsaturated aldehydes, and hydroxyl-, keto-, and epoxyhydroxy fatty acid derivatives. They are known for their broad cytotoxicity that targets cytoskeleton, calcium signalling and cell death pathways [13,52]. Recently, two apoptosis-inducing galactolipids were isolated from the marine diatom *Phaeodactylum tricornutum* [36], and their apoptogenic effects were tested on mammalian cell lines. However, the apoptogenic effect from the as yet unknown compound of *Cocconeis* that leads to female sex is restricted to the androgenic gland, and is effective only in a short life stage of the shrimp.

In addition, it is rather unlikely that the apoptotic effects are caused by unsaturated straight chain aldehydes, because these molecules would have an absorption in the UV region and are volatile. This supports observations with planktonic marine diatoms that have a high capacity of aldehyde formation from polyunsaturated fatty acids but do not induce apoptotic reaction in the AG of *H. inermis* [53]. Thus, the bioactive principle must be different from the aldehydes that reduced egg-hatching rates, inhibited cleavage of sea urchin embryos, or arrested growth in CaCo2 cell lines [37].

In our case, although all bioassays confirmed the activity of fraction 4d, the presence of chlorophylls and other metabolites normally abundant in plant cells prevented an easy detection of the active compound that might have a promising apoptogenic activity. For this reason, a different fractionation method (Bond Elut C18, Varian) was applied in the fifth separation. In fact, this method allowed for pooling most plant metabolites in the first eluates, while maximizing the separation of other bioactive compounds. The fifth bioassay gave very clear results, indicating the compound was divided into two parts (as previously observed in the fourth bioassay), *i.e*., a relatively polar compound, present in eluate A, and a highly lipophilic compound maximizing into the eluate C. According to the bioassays 1–4, these two compounds could correspond to the ones separated into the fractions 1-2 and 4d, respectively.

It is also interesting to observe that the two mixtures of compounds are solved by different solvents: The activity of eluate A is at a maximum after solution in methanol, while the activity of eluate C is at its maximum in acetonitrile. This confirms the non-polar properties of the compounds present in eluate C, apparently corresponding to the ones previously separated in the fraction 4d.

For this reason, considering the higher stability and consistency of bio-activity of the fraction 4d in respect to more polar fractions, we decided to focus on the compounds present in this non-polar eluate, fractionating it into five parts (fractions C1–C5 in Figure 7b). The results are remarkable, indicating the peak of activity was determined by fraction C3. It likely contained a single compound (single UV-C peak), not corresponding to any standard already known. Thus, its structural elucidation is required.

## 4. Experimental Section

### 4.1. Diatom Isolation

Diatoms were collected by deploying metal panels painted with low-adhesion silicon coatings in the harbour of Ischia (Bay of Naples, Naples, Italy; Figure 1c) for 30 days, in April, when most species of *Cocconeis* produce natural blooms. Small amounts of micro-algae were scraped off the panels using a cover slide, and they were then cultivated in 3 mL plastic multi-wells in *f/2* medium [54]. Diatoms were selected by means of a Leica micro-manipulator to obtain several mono-clonal cultures of *Cocconeis scutellum parva*. Mother cultures were then identified to the species level by SEM observations.

### 4.2. Diatom Culture and Harvest of *Cocconeis* Biomass

The cells growing on six mother-culture plastic dishes (4 cm) were collected by a Pasteur pipette, 15 days after inoculation, when the surface was almost completely covered by diatoms. The obtained diatoms were pooled in a sterilized beaker, and the suspension was divided into 20 parts and subsequently transferred to 20 Petri dishes (7 cm diameter) filled with *f/2* medium. These dishes were kept in a thermostatic chamber for 15 days to obtain a diatom biofilm. After 15 days, the surfaces of the Petri dishes were covered with diatom biofilms, which were collected and pooled in a sterilized beaker. One mL of this suspension was inoculated into each of the 200 larger Petri dishes (14 cm diameter) containing the *f/2* medium. The diatoms were cultured for 15 days in the same thermostatic chamber. After removal of the medium, the dishes were quickly rinsed twice with distilled water, frozen at −20 °C and then lyophilized overnight. The dried diatoms were scraped off by a blade, weighed and kept in dry vessels at −20 °C until extraction. All the transfer operations were performed under a laminar flow hood and the dishes and laboratory instruments were previously autoclaved at 120 °C.

### 4.3. Extraction and Fractionation

The search for the bioactive compound was conducted during six subsequent years. This time was necessary because the shrimp’s spawning season allowed only one bioassay per year and each new production of diatom biomasses, for the subsequent extractions, required various months. After every bioassay-guided fractionation, with subsequent bioassay, HPLC separations were optimized based on the new information obtained, as reported in the following paragraphs and summarized in Table 1.

**Table 1:** Summary of the fractionation procedure followed during six subsequent years, indicating the biomass analysed, the separating procedure and the total number of fractions collected to perform subsequent bioassays, as reported in the figures of this paper.

Sep. Nr.	Year	Biomass separated (by HPLC or C18 cartridge)	Extraction solvent	Separation procedure	Fractions collected	Figure
1	2008	250 mg *Cocconeis*	80% ACN	HPLC; C18 analyt ^1^	1-2-3-4-5 ^3^	2
2	2009	250 mg *Cocconeis*	80% ACN	HPLC; C18 analyt ^1^	2-3-4a-4b-4c-4d-5 ^3^	3
3	2010	300 mg *Cocconeis*	80% ACN	HPLC; C18 analyt ^1^	1-2a-2b-2c-2d-4a-4b-4c-4d-5 ^3^	4
4	2011	300 mg *Cocconeis*	80% ACN	HPLC; C18 analyt ^1^	1-2a-2b-2c-2d-4a-4b-4c-4d-5 ^3,4^	5
5	2012	300 mg *Cocconeis*	Ca. 80% ACN	C18 Bond Elut Cartridge	Eluate A,B,C ^3,5^	6
6	2013	560 mg Eluate C	80% ACN	HPLC; C18 analyt ^2^	C1-C2-C3-C4-C5 ^6^	7

^1^ linear gradient of 100% solvent A (80/20, v/v, MeOH/H_2_O) to 100% solvent B (80/20, v/v, MeOH/acetone) in 10 min, then solvent B for 20 min; ^2^ isocratic elution with 90% aqueous ACN; ^3^ fractions/eluates were dissolved in 100% MeOH and added to food particles for the bioassays; ^4^ fraction 4d was additionally reconstituted in ACN; ^5^ eluates were additionally reconstituted in ACN; ^6^ eluate C sub-fractions were dissolved in ACN only.

#### 4.3.1. First Separation

Two hundred and fifty mg of freeze-dried biomass of *C. scutellum parva* were extracted with 12.5 mL aqueous 80% acetonitrile. The soluble part was separated from the insoluble by centrifugation (10 min) in Corex glass tubes at 7400× *g* (DuPont Instruments, Newtown, CT, USA). The extract (equivalent to 200 mg of dry biomass) was separated into five fractions on an analytical C18 reversed phase HPLC column (GromSil ODS 4 HE, 250 × 4.6 mm, 5 µm particle size; Stagroma, Germany; 1 mL/min flow rate; 30 °C oven temperature; diode array detection) using a linear gradient of solvent A (80/20, v/v, MeOH/H_2_O) and solvent B (80/20, v/v, MeOH/acetone). The time program was: Solvent A, 0% to 100% in 10 min, then solvent B, 100% for 20 min. The shift of the retention time of the eluted compounds between the first and the last separation was 0.1 min. The fractions were evaporated on a Rotavapor (Büchi, Switzerland) at 40 °C and 30 mbar. The residues were taken up in 5 mL 100% MeOH and transferred into glass vials. The solvent was evaporated in a nitrogen gas stream and the dry residues were stored at −20 °C. The equivalent of 200 mg *Cocconeis* extract were used in the bioassay.

#### 4.3.2. Second Separation

An identical procedure, as above, was applied. In addition, since high bioactivity was found in fraction 4 previously tested, this was further divided into smaller fractions (4a–d).

#### 4.3.3. Third Separation

At this stage we applied the same separation procedure as described above, but we increased the number of fractions, in order to determine more precisely the elution time of the bioactive compounds. In this case, we separated 300 mg of freeze-dried *C. scutellum parva*, and used 250 mg in the bioassays. Fraction 2 was further divided into four sub-fractions (2a–d), while fraction 3 was included into fraction 4a.

#### 4.3.4. Fourth Separation

In this experiment the same separation and fractionation as reported above (third separation) was performed to verify the results of the previous year.

#### 4.3.5. Fifth Separation

Three hundred mg of freeze-dried *C. scutellum parva* were gently potterized for 2 min in 6 mL of water (deionized ultra-pure water). The suspension was left at room temperature for 10 minutes, which allowed the action of wound-activated enzymes. After a transfer into a 50 mL centrifuge tube, acetonitrile was added in the ratio 1:10 (mL:mg) and incubated for 2 h in the dark. After centrifugation (4000 rpm, 10 min; Centrifuge Heraeus Christ GmpH, Hanau, Germany) at room temperature, the supernatant was collected for further separation on a cartridge (Varian Bond Elut C18, Agilent Technologies, Basel, Switzerland) applying slight vacuum. The cartridge was cleaned and equilibrated by using one volume (60 mL) of 30%, 50%, 80%, 100% and finally 30% aqueous ACN. The cartridge was never allowed to get dry during the passage. Ten mL of the diatom extract was combined with 16.6 mL H_2_O, thoroughly mixed and passed through the equilibrated cartridge. Shortly before becoming dry, 33.4 mL of 30% aqueous ACN was added. Thus, eluate A had a final concentration of 10% ACN. In a second step, the cartridge was eluted with three volumes of 80% ACN (180 mL) to remove the green chlorophyll derivatives (eluate B). Finally, the cartridge was eluted with two volumes of 100% ACN (120 mL) to get the eluate C. The low-boiling organic solvent ACN was removed on a rotary evaporator Rotavapor R-200, Büchi (Newcastle, DE, USA), and subsequently the water was removed using a Liophilizator Lio5P, 5Pascal (Milan, Italy) until dry residues were obtained. The residues were dissolved in 5 mL ACN and transferred into smaller glass vials. After removal of the solvent (Rotavapor) the dried extracts were stored at −20 °C until further use. The equivalent of 300 mg *Cocconeis* extract were used in the bioassay.

#### 4.3.6. Sixth Separation

Since the bioactive compound seemed to have no important absorption in the long-wave UV, we changed the solvents used for separation and applied isocratic conditions. Acetonitrile and water, which allowed seeing UV-spectra in the UV-C range, were used for further analyses. Eluate C (ACN) was chosen for further analyses, according to the results of bioassays. Eluate C (equivalent to 560 mg freeze dried biomass of *C. scutellum parva*) was dissolved into 2 mL 80% aqueous acetonitrile. The extract was separated into five fractions on an analytical C18 reversed phase HPLC column GromSil ODS4 HE, Stagroma GmbH (Reinach, Switzerland) 250 × 4.6 mm; 5 µm particle size; 1 mL/min flow rate; 30 °C oven temperature; diode array detection). Solvent A was UV-treated H_2_O, while solvent B was acetonitrile. All separations (20 runs) were done with 90% B under isocractic conditions. The peak (195 nm) eluting at 19.8 min was chosen as a possible bioactive candidate due to former separation procedures and it was collected into a single fraction. The equivalent of 300 mg biomass was tested in the bioassays.

### 4.4. Bioassay Procedure

For the bioassays we followed the techniques described by Zupo and Messina [15]. The seawater used for the experiments was filtered overnight through a mechanical absorbent filter 250 Classic, Eheim GmbH (Deizisau, Germany) containing perlon wool and activated charcoal. The water was treated with ozone for 4 h and aerated 4 h prior to be used for larval cultures.

Ovigerous females of *Hippolyte inermis* were collected in a *Posidonia oceanica* meadow off Lacco Ameno (Island of Ischia, Naples, Italy), sorted on boat and kept in plastic bags up to the return in the laboratory. The ovigerous females were then transferred individually into 2 L conical flasks containing about 1.8 L of filtered seawater, along with a small portion of *Posidonia* leaf, for shelter, in a thermostatic chamber maintained at 18 °C, with Gro-Lux fluorescent tubes at a mean irradiance of 250 µmol m^−2^ s^−1^ ten hours a day. After some days, each female released larvae (from 20 to 400) that were collected and kept for our bioassays. Their mothers were immediately returned to the sea. The larvae collected were transferred in groups of 80 in 1 L conical flasks containing approximately 800 mL of clean filtered seawater, kept in the same thermostatic chamber as described above.

Larvae were fed by adding four *Artemia salina* nauplii and four *Brachionus plicatilis* individuals per mL of seawater, for the first 7 days. During the following days, the administration of *Brachionus* was interrupted and *Artemia nauplii* were enriched using an Algamac, Biomarine (Hawthorne, CA, USA) integrator [15]. After about 30 days the larvae settled and they were transferred in groups of 25 post-larvae into 14 cm (diameter) vessels containing 400 mL of filtered seawater. They were fed a composed food made of three items in equal proportions: SHG “*Artemia* Enriched”, SHG “Microperle” and SHG “Pure *Spirulina*”. This is the composition of the basic food used for our negative controls. To test the activity of *C. scutellum parva*, its dried extracts were dissolved in 2 mL of methanol (MeOH) and mixed to the prepared composed food. The solvent was then evaporated by a rotary evaporator to obtain the dried foods supplemented with the diatom fractions.

To prepare the positive controls, dried diatoms (*C. scutellum parva* of identical batches) were added to the basic food in a ratio of 2:1 (w/w). All foods were prepared at the start of the experiment (the first day of post-larval growth) and kept in a refrigerator at −20 °C up to the administration. Dry food (5 mg) was offered daily in each vessel containing 25 post-larvae of *H. inermis*.

In detail, we assayed three replicates (25 individuals each) for each negative and positive control, as well as three replicates (25 individuals) for each fraction to be tested. The daily mortality was measured by collecting and counting larvae by a Pasteur pipette, while water and food were replaced. Post-larvae were sacrificed after 40 days, fixed in 70% ethanol, and subsequently observed under the dissecting microscope to measure their total body length using millimetric paper and a metal bar. Their second pleopods were collected on a slide and analysed under the optical microscope to check the presence or absence of a masculine appendix, indicating male or female sex, respectively.

Each bioassay tested the activity of further fractions (as above reported) against positive and negative controls. In particular, the first bioassay tested the activity of five fractions obtained from the first separation. In addition, we measured the activity of the “residual”, consisting of frustules and broken diatoms remained in a pellet after the centrifugation of the extraction solvents. This test was aimed at checking if the chosen solvents effectively extracted all active compounds.

The second bioassay tested the activity of seven fractions obtained from the second separation, as reported previously. Also in this case the activity of the “residual” was checked in order to exclude that the active compound was still present in the solid pellet after centrifugation.

The third bioassay tested the activity of 10 fractions obtained from the third separation. The fourth bioassay tested again the activity of 10 fractions obtained from the fourth separation to confirm the data of the previous one. In addition, we measured the activity of the fraction 4d twice. The first test was performed by adding 2 mL of methanol (667 µL for three times) to the vessel containing the dried fraction 4d. It was vigorously shaken and added to the dry food. For the second test, the same vessel was refilled with 2 mL of acetonitrile (667 µL for three times) and the procedure was repeated. In this way, we aimed at checking if the solvent used to transfer the fractions to the experimental foods (methanol) was able to completely dissolve the active compound in the lipophilic fraction 4d.

The fifth bioassay was performed on three eluates obtained with a different separation method, as reported previously. In this case, all tests were repeated twice: The first time the eluates were dissolved and transferred to the dry foods using 2 mL of methanol. The second time, the same vessels were refilled with 2 mL of acetonitrile, then transferred to dry foods and evaporated. In this way we wanted to test the efficacy of methanol in dissolving completely the active compound present in the dry film covering the vessels.

The last bioassay was performed on five fractions of the eluate C (sixth separation), reconstituted in acetonitrile, as indicated above.

### 4.5. Statistical Analyses

The percentage of females normalized to the total number of mature individuals (F/mat%) was calculated for each test. This index permits to determine the effect of apoptogenic compounds on the sex of mature shrimps, avoiding the influence of individuals that, at the end of the experiment, were still immature. The significance of differences between each treatment and the controls was tested by applying the *Z*-Test on proportions (STATS 2002 v. 2.7) for the observed F/mat% values.

## 5. Conclusions

A strict bioassay-controlled separation was performed in the present investigation and led to the characterization of bioactive fractions. Freeze-dried biomasses of *C. scutellum parva* were extracted with 80% aqueous ACN, and all the activity was found in the extract. Currently, the structure elucidation of the bioactive compound is in progress; however, time is needed to obtain a sufficient amount for spectroscopic analyses, such as HR-MS and NMR. Future research will test the activity of the pure compound on our model shrimp to confirm the identity of the apoptogenic compound. This confirmation, in fact, will disclose new challenges, since the comprehension of the mechanism of action on the cell machinery [55,56] could indicate the factors underlying the specificity of the apoptotic process [34], leading to further important medical applications.
